# Factors Influencing Physicians’ Continuous Blogging: A Survey

**DOI:** 10.3390/healthcare9080958

**Published:** 2021-07-29

**Authors:** I-Chiu Chang, Kuei-Chen Cheng, Hui-Mei Hsu, David C. Yen

**Affiliations:** 1 Department of Information Management, National Chung Cheng University, Chiayi County 62102, Taiwan; misicc@mis.ccu.edu.tw (I.-C.C.); Kccheng1961@gmail.com (K.-C.C.); 2 Department of Business Management, National Kaohsiung Normal University, Kaohsiung City 80201, Taiwan; hmhsu@nknu.edu.tw; 3 Jesse H. Jones School of Business, Texas Southern University, Houston, TX 77004, USA

**Keywords:** physicians, blogging, social cognitive theory

## Abstract

Background: Health information can be more easily transmitted and diffused through the Internet, but questionable online health information often misinforms patients. Physicians have a duty to inform patients how to achieve positive health outcomes. Many physicians often write blogs to provide patients with the right health information. However, most articles available on this subject only describe the blog phenomena without providing a theoretical background and an empirical analysis of doctors using blogs. Methods: This study based on social cognitive theory (SCT) explores the factors influencing physicians’ intention of continuously blogging. A total of 887 physician bloggers were invited to participate in an online survey and 128 valid responses were received. Results: The SCT was proven to be useful in explaining 36.8% of the variation in physicians’ continuous intention to blog. Conclusions: We provide references for platform developers with different strategies to motivate doctors to blog, and the implications and limitations of this study are discussed.

## 1. Background

The Internet offers widespread access to health information and millions of ‘‘cyberchondriacs’’ claim to have gone online to obtain health information [1]. However, questionable online health information often misinforms patients and has led them to confusion, distress, or an inclination towards detrimental self-treatment and/or misconceptions about particular diseases [2]. Research shows that persons who are older, have lower education levels and with lower internet skills generally use a health care professional as their primary source of health information [3]. While physicians still remain as the most trusted information source for patients, physicians going online can benefit more people in many ways. Especially, useful communication between physicians and patients helps to address chronic diseases more effectively and efficiently [4], and lowers the treatment burden [5]. 

US physicians widely employ Internet-based communication technologies [6], such as email and weblogs for further reengineering their various interactions with other people or patients. Running a weblog involves needing to frequently modify the web pages with interactive and dynamic features. It is a time-consuming task. Commercial businesses regularly create content to inform and educate their customers through blogs [7]. While lack of time was found to be one of the main barriers to IT adoption for physicians, physician bloggers perhaps hope to propagate the correct medical knowledge/information and ways to protect against diseases through blog writing.

Since December 2019, following the ongoing outbreak of the coronavirus disease COVID-19 all over the globe, people have acquired and exchanged various types of information at a historic and unprecedented scale using social media (e.g., Facebook, Twitter, etc.). Meanwhile, people have also acquired a great deal of misleading rumors and conspiracy theories that have caused panic, racism, and mass hoarding of materials (such as surgical masks). The World Health Organization identified in its Novel Coronavirus (2019-nCoV) Situation Report-13 that “the 2019-nCoV outbreak and response has been accompanied by a massive ‘infodemic’—an over-abundance of information—some accurate and some not—that makes it hard for people to find trustworthy sources and reliable guidance.” [8]. Merchant and Lurie indicated that “Directing People to Trusted Sources” and “Counteracting Misinformation” through integrating social media are critical in managing the current pandemic as well as transforming different aspects of preparedness and responses for the future [9]. In the UK, a group of diabetes doctors wanted to provide “a credible source of guidance during what has fast become an epidemic of misinformation.”, so they have set up a social media account to provide them with “a secure base” of information, and further reduce people’s anxieties and promote psychological resilience towards COVID-19 [10]. 

Many Taiwanese physicians are also blogging. The Taiwan healthcare services were rated first worldwide by TheRichest.com (accessed on 14 August 2019) [11] and second worldwide by the Economist Intelligence Unit [12]. The World Health Organization (WHO) ranked Taiwan National Healthcare Insurance (NHI)’s fairness as second worldwide in its financial contribution index [13]. Enrollment in this insurance system has risen continuously and currently it includes around 99.6% of the population [14]. However, this insurance induces medical demand. According to the NHI database, the average number of physician visits per capita in 2017 was 16.9 [15]. Under such a heavy workload and the aforementioned lack of time for IT adoption, understanding the value that physicians expect to get from blogging is worth researching.

The motivation of the general public to blog may include to document their lives, to release emotional stress, to express opinions to the public, to muse by writing, to support a community forum of interest, for their enjoyment, and to share knowledge. A survey by DocStyles found that about 12.9% of physicians in the US have written a blog [6]. Besides incentives such as marketing and providing advice [16], Kovic et al. suggested that there are three other motivations for physicians to blog: sharing their practical knowledge or skills with others, influencing the way others think, and expressing oneself creatively [17]. Many studies concluded that sharing medical information and giving advice are both important motivations for physicians to blog [18,19,20].

However, most articles available on this subject only describe these phenomena without providing a theoretical background and an empirical analysis of the phenomena. To bridge this gap, the often-used social cognitive theory (SCT) was thus adopted as the theoretical base in this study to explore the influencing factors that can further motivate physicians to continue blogging and a survey was conducted to validate the proposed research framework.

### 1.1. Theoretical Background

SCT favors conception of interaction based on triadic reciprocity, which includes environmental influences, cognitive and other personal factors, and behavior [21]. Two main determinants that affect person’s cognition are self-efficacy and outcome expectations [22].

Beliefs of personal efficacy influence people’s goals and their aspirations. People tend to shun tasks that exceed their capabilities and approach situations that they are capable of handling [23]. The stronger their perceived self-efficacy, the higher the goals people set for themselves, leading to a firmer commitment. Self-efficacy beliefs generate the outcomes that people expect from their efforts [24], a judgment of the consequences of a certain behavior. Outcome expectations when using an Information System (IS) fall under “perceived usefulness” in the technology acceptance model (TAM) [25]. The origins of outcome expectations of the SCT took several forms with both positive and negative effects [21]. Most IS studies excluded the effects of negative outcome expectations. Since the outcome of blogging may be either positive or negative, applying SCT in blogging settings may verify the SCT’s applicability and remedy the knowledge gap addressed in the IS research area. In other words, by testing the negative outcome expectation of the SCT in the blogging setting, this study can further verify the applicability of the original SCT in the IS area. 

### 1.2. Research Hypotheses

Blogging is a voluntary personal behavior and the outcome of blogging may vary and be quite complicated. Specifically, negative outcome expectations of blogging, such as negative social outcome expectations and negative status outcome expectations, have been proposed [26]. Therefore, both positive outcome expectation constructs from the IS literature and the original negative outcome expectation of SCT were adopted to generate Hypothesis 1, as follows:

**Hypothesis** **1** **(H1a).** *The higher the self-efficacy a physician perceives, the higher the positive performance outcome expectations of blogging he/she displays.*

**Hypothesis** **1** **(H1b).** *The higher the self-efficacy a physician perceives, the higher the positive personal outcome expectations of blogging he/she displays.*

**Hypothesis** **1** **(H1c).** *The higher the self-efficacy a physician perceives, the lower the negative outcome expectations of blogging he/she displays.*

Further, the relationship between the outcome expectations and target behavior intention was found to be rather mixed and it also lacked consistency in prior studies [27]. Therefore, it is worthwhile to investigate and analyze this relationship in a blog setting. As per the above discussion, the outcome of blogging could be positive or negative, and consequently the Hypothesis 2 was developed as below:

**Hypothesis** **2** **(H2a).** *The higher the positive performance outcome expectations of blogging a physician displays, the greater the extent to which he/she would like to blog continuously.*

**Hypothesis** **2** **(H2b).** *The higher the positive personal outcome expectations of blogging a physician displays, the greater the extent to which he/she would like to blog continuously.*

**Hypothesis** **2** **(H2c).** *The higher the negative outcome expectations of blogging a physician displays, the lesser the extent to which he/she would like to blog continuously.*

The outcome expectations may have a positive relationship with satisfaction. Therefore, the Hypothesis 3 was proposed to clarify this confusion:

**Hypothesis** **3** **(H3a).** *The higher the positive performance outcome expectations of blogging a physician displays, the greater the satisfaction from blogging he/she would feel.*

**Hypothesis** **3** **(H3b).** *The higher the positive personal outcome expectations of blogging a physician displays, the greater the satisfaction from blogging he/she would feel.*

**Hypothesis** **3** **(H3c).** *The higher the negative outcome expectations of blogging a physician displays, the lesser the satisfaction from blogging he/she would feel.*

Both satisfaction from IS usage and the intention to continue to use the IS are regarded as important indicators of the IS success. The results of prior studies show that satisfaction has a positive impact on the intention to continue to use [28,29,30,31,32,33]. Therefore, the last hypothesis was proposed as follows.

**Hypothesis** **4** **(H4).** *The higher the satisfaction a physician feels while blogging, the greater the extent to which he/she would like to continue to blog.*

## 2. Methods

### 2.1. Data Collection

Ethical approval for the study was obtained from the Joint Institutional Review Board at Tungs’ Taichung MetroHarbor Hospital. Physicians who had created a blog on Taiwan’s popular blog service providers (Wretch, Yahoo, Pixnet, Yam, Udn, Google blogger, and Kingnet) were the subjects in this study. Furthermore, we used the search engine provided by the aforementioned blog platforms to search for physicians’ blogs and filter out blogs of the veterinarians, pseudo-doctors, and unverified doctors where the contents of a blog did not include professional medical information or advice. In total, 877 appropriate physician bloggers were identified.

An online questionnaire survey was used to collect data. Either the URL of online questionnaire was sent to the physician bloggers via email or a private invitation message was posted on their blogs. By doing so, only the invited physician bloggers could access the online questionnaire. Our data collection process was anonymous. A cover letter explained the research goal and questionnaire to the participants and a statement of “By completing this survey, you are consenting to participate in this study” was placed in the beginning of the questionnaire to obtain participants’ consent.

In order to enhance the response rate, an incentive gift was provided to each respondent who submitted a valid questionnaire. 

### 2.2. Measurement

The research model comprised six constructs: blogger self-efficacy, positive performance outcome expectation, personal outcome expectation, negative outcome expectation, satisfaction, and continue to blog intention, with the measurement items derived from prior research. All variables are scored on a 5-point Likert scale, where 1 represents “strongly disagree” and 5 represents “strongly agree” in the questionnaire except for “satisfaction”. The measurement items of blogger self-efficacy were adapted from Liu’s research [34], while measurement items of outcome expectations were derived from the studies of Liu [26] and other research related to blogging motivations [16,17]. In total, 16 positive items and 7 negative outcome expectations were adapted, such as blogger self-efficacy (3 items), positive performance outcome expectation (5 items), positive personal outcome expectation (5 items), negative outcome expectation (3 items), satisfaction (4 items), and continuous intention to blog (3 items). The measurement items of satisfaction and continue blogging intention were derived from Bhattacherjee’s study of extending the IT continuance model [35]. The scale of satisfaction was measured on a 5-point Likert scale and anchored among four semantically different adjective pairs from 1 to 5 including “very dissatisfied/very satisfied”, “very displeased/very pleased”, “very frustrated/very contented”, and “absolutely terrible/absolutely delighted.” An expert panel consisting of four professors and three experienced directors working in the informatics field was formed to review the appropriateness of the items, and the format, length, and wording of the scales. Revisions of the questionnaire were done based on suggestions of the expert panel. Details of questionnaire items are shown in Supplementary Materials Table S1.

### 2.3. Participants

After removing incomplete questionnaire, we collected a total of 128 valid online questionnaires. Of all the participants, male physician bloggers were the overwhelming majority (82.0%). The youngest respondent was 27 years old while the oldest one was 71. Their ages were distributed into different groups, with the largest group being 30–39 years old (46.1%). Most physician bloggers could be identified easily in their blogs by identification photograph(s) (70.3%), their real name (63.3%), and by providing other personal information such as their age, institution, and work schedule of practice (42.2%). The top five specialties that physician bloggers practice in were family medicine, obstetrics and gynecology, surgery, ENT (ear, nose, and throat), and dermatology. Most of the physician bloggers were visiting staff (V.S.) (77.3%) and the resident group (R1-R4) (14.8%), and practiced in medical centers (40.6%) and clinics (33.6%). Details are provided in Table 1.

## 3. Results

### 3.1. The Motivations of Physicians and Their Blog Contents

A very large majority (91.4%) of the physicians created a personal blog voluntarily and 82.0% maintained their blogs themselves, without commissioning others to handle their blogging. The top five self-reported motivations to create a blog in order from high to low were to “share medical knowledge with the public and medical staff”, “record my life”, “provide medical consultations for the public”, “express my feelings”, and finally “share information not related to medical knowledge.” The content of the doctors’ writing in the blogosphere was rich in knowledge and different in style and taste. Most doctors (89.8%) provided “medical knowledge” content on their blogs. “Travel experience” sharing ranked second (50.0%), and the third one was a tie between “joys and sorrows of job” and “literature creation” with 47.7%. Details are summarized in Table 2.

### 3.2. Measurement Model Testing

Partial least square (PLS) is a component-based structural equation modeling (SEM) method and can be used for theoretical confirmation. PLS maximizes the explained variance of endogenous latent variables by estimating partial model relationships in an iterative sequence of ordinary least squares (OLS) regressions [36]. It is a powerful tool because of its minimal demands on measurement scale, sample size, and residual distribution [37]. SmartPLS 2.0 M3 was used to test our model. The convergent validity and discriminant validity as well as construct validity were examined. A rule of thumb suggested by Barclay et al. [38] for convergent validity with many researchers’ acceptance is that factor loading of each item should be greater than 0.7 and have a significant t-value (*t*-value > 1.96, *p* < 0.05) [37,38,39] to be acceptable. In addition, composite reliability (CR) was calculated as the internal consistency of a construct in PLS. The CR value was suggested to be 0.7 as a benchmark by Nannally [39]. In this study, the CR value of each construct was higher than 0.8, but some measurement items were discarded because of lower loading values (under 0.707) at an initial confirmatory factor analysis. Details are provided in Table 3.

Since the research model and questionnaire items were adapted from prior related studies, face validity was verified by the expert panel. Regarding discriminant validity, three criteria were used: (1) the load of each measurement item should be at the highest theoretical construct, and be unlikely to involve other constructs [37]; (2) Average variance extracted (AVE) explains the variance captured by a latent variable and therefore, AVE should not be less than 0.5 [40]; and (3) the square of root of AVE should be larger than the correlation among the specific construct and any other constructs [37]. The obtained results showed that all constructs met the above criteria of discriminant validity (Table 3 and Table 4).

### 3.3. Structural Model Testing

**Figure 1:**
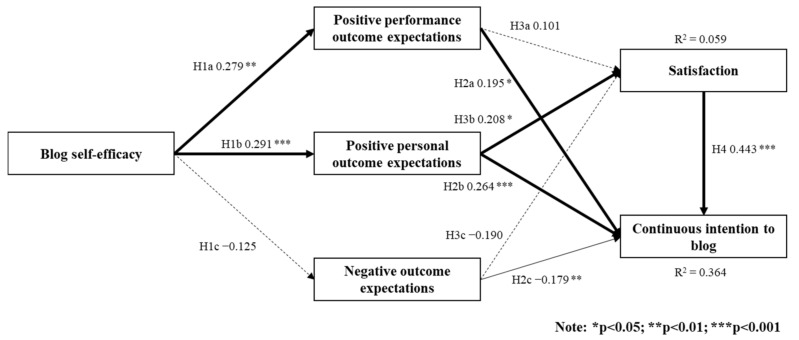
PLS analysis results of the structural model test.

Analysis of the structural modeling test was completed in two steps. First, estimation of parameters in the inner and outer model was by PLS path modeling with a path weighting scheme. Secondly, the significance of path coefficients was then examined by a bootstrapping technique with 500 times of random re-sampling approach. Figure 1 depicts the results of the structural modeling test. 

The R square is a measure of a model’s predictive accuracy, and it represents the exogenous variables’ combined effect on the endogenous variables; this effect ranges from 0 to 1 [41]. Chin [37] describes R square values of 0.67, 0.33, and 0.19 in path models as substantial, moderate, and weak, respectively. Hair et al. [42] indicated that acceptable R square levels depend on the research context.

The R square of the continued intention to blog was 0.364. Physicians’ blog self-efficacy had a significant impact on both positive performance and personal outcome expectation of blogs and a non-significant effect on negative outcome expectation. Therefore, Hypothesis 1a,b were supported, but Hypothesis 1c was not supported. Regarding the relationships between physicians’ outcome expectations of blogs and continued intention to blog, all types of outcome expectations affected the continued intention to blog significantly. Therefore, Hypotheses 2a–c were all supported. Other than the personal outcome expectation, the other two outcome expectations had no significant effects on blogger satisfaction. As a result, only Hypothesis 3b was supported. Lastly, blogger satisfaction was found to have a significant impact on the intention to continue to blog and therefore Hypothesis 4 was supported. 

## 4. Discussions

### 4.1. Analysis of the Findings

This study used Bandura’s argument and incorporated both positive and negative outcome expectations in physicians’ expectations of blogs. The results conform to the SCT in that outcome expectations influence doctors’ continued blogging behavior and hence, partially support Bandura’s argument in that blog self-efficacy affects only the positive outcome expectations and has no impact on the negative outcome expectations. 

The “positive performance outcome expectation” construct was employed as the outcome expectation related to the doctors’ jobs. Many doctors shared medical knowledge or information, communicated with the readers or their patients, or even marketed themselves through their blogs. Therefore, higher positive expectations obviously motivate physicians’ intention to blog continuously and the results confirm prior studies [27]. Furthermore, physicians’ personal outcome expectations had higher impacts on their intention to blog continuously than performance outcome expectations with impact coefficients of 0.30 to 0.23, respectively, which totally fulfilled the original purposes of blogging. 

Lee and LaRose revealed that self-efficacy had no impact on negative outcome expectations of embarrassment for disclosure descriptive information of web-based personal health records [12]. Since most information on doctor blogs is descriptive information, this study confirmed the findings of Lee and Larose. However, the negative outcome expectation of physicians for blogging was low (mean = 2.33), which means most physicians perceive that blogging behavior brings little negative outcomes, regardless of perceived blog self-efficacy being high or low.

In previous research, the relationship between outcome expectations and satisfaction was found to be inconsistent. The findings of this study show that only the personal outcome expectations have significant effects on blogger satisfaction. The possible explanation for this may be that the other two expected outcomes were not realized yet, since a blog has to be run for a long time before it yields some decisive results. The low R square value for satisfaction means that some other constructs as mediators or moderators might be needed to explore the effects on satisfaction in future studies. The research model based on SCT accounts for 36.4% of the variance in physicians’ continued intention to blog, which indicates the reasonable research value of the SCT. However, other important factors might be missing, since the research model and questionnaire items were adapted from prior related studies.

### 4.2. Research Limitations

The use of a cross sectional study and survey method resulted in the following limitations. Firstly, blogging is a continuous behavior, thus some responses might be fallacious with recall bias due to a vague memory recollection while answering the questionnaire. Secondly, the questionnaire was self-reported by respondents and it is hard to confirm the authenticity of respondents’ answers; perhaps only those who favored the subject issue might have returned the questionnaire. Some bloggers may have not identified themselves as physicians and were therefore not recruited for this study. Meanwhile, physicians might have the halo effect of the positive blogging effect and have biased opinions about the negative impacts of blogging. Finally, caution may be needed while applying the results of this study in different countries, since different factors in different cultures may affect physicians’ behavior intentions differently.

## 5. Conclusions

This study proves that the SCT is useful in explaining the affecting factors of physicians’ continuous blogging intention. Both physicians’ positive and negative outcome expectation of blogging impact their continuous intention to blog. Since most prior research only explored the effects of positive outcome expectation of using information systems (IS), the results of this study fill the knowledge gap by applying social cognitive theory to the research area of IS usage. As discussed earlier, insurance induces increased medical demand and may impact physicians’ work load and quality of healthcare services. Alternatively, when physicians do not have enough time for patients during their visits, their blogs may serve as an additional channel to communicate and save medical resources. Professional or governmental sites are useful to provide solid and accurate health information to fulfill public needs of acquiring knowledge and managing disease.

For future studies, important healthcare concerns other than those from prior research in IT/IS field might need to be explored; in particular, as COVID-19 becomes more and more serious, physicians’ blogs may become a critical platform for pandemic preparedness and responses. Physicians can propagate the correct medical knowledge / information and ways of protecting against the coronavirus through blogs to reduce the spread of the pandemic. Utilization of interactive blogging features can certainly increase much-needed transparency and enhance the depth of information [43]. Furthermore, blog contents may include the physician’s personal information, which may create a warmer physician-patient relationship and make the disease co-management task easier.

Helping others is a creed for physicians, including those who seek help from the Internet. Using the blog as a vehicle to educate, heal, and prevent disease is coincident with the physicians’ oath. Therefore, this study strongly recommends that physicians create blogs to communicate with the public and blog continuously. To motivate physicians to continue to blog, the platform developers can encourage physicians by equipping them to have higher blog self-efficacy, a high positive performance/personal outcome expectation and lower negative outcome expectation from blogging, and also a higher satisfaction with their blogging behavior.

## Figures and Tables

**Table 1:** The Demographic Description of Samples.

Items	*n*	%	Items	*n*	%
Gender			Specialty		
Male	105	82.0%	Family Medicine	12	9.4%
Female	23	18.0%	Obstetrics and Gynecology	11	8.6%
Age group			Dermatology	10	7.8%
Under 30	13	10.2%	E.N.T.	10	7.8%
30–39	59	46.1%	Surgery	10	7.8%
40–49	38	29.6%	Pediatrics Medicine	8	6.3%
50- and above	19	14.9%	Ophthalmology	6	4.7%
Career Level			Internal Medicine	5	3.9%
Visiting Staff	99	77.3%	Emergency Medicine	4	3.1%
Fellow	2	1.6%	Orthopedics	3	2.3 %
Chief Resident	8	6.3%	Plastic Surgery	3	2.3%
Resident	19	14.8%	Neurology	2	1.6%
Practice Institution			Neurology Surgery	2	1.6%
Medical center	52	40.6%	Psychiatry	2	1.6%
Regional hospital	27	21.1%	Radiology Diagnostic	2	1.6%
District hospital	11	8.6%	Rehabilitation	2	1.6%
Clinics	43	33.6%	Cardiovascular Medicine	2	1.6%
License			Anesthesiology	1	0.8%
West Medicine	100	78.1%	Cardiovascular Surgery	1	0.8%
Chinese Medicine	19	14.8%	Chest Medicine	1	0.8%
Dentist	5	3.9%	Gastrointestinal Medicine	1	0.8%
West & Chinese Medicine	4	3.1%	Geriatrics	1	0.8%
Identity			Infection	1	0.8%
Real full name	81	63.3%	Neurology	1	0.8%
Real last or first name	18	14.1%	Nuclear Medicine	1	0.8%
Fake name	29	22.7%	Urology	1	0.8%
Identifiable photograph	90	70.3%			
Hard to identify photo	6	4.7%			
Fake or no photo	32	25.0%			

**Table 2:** Physicians’ Motivations to Blog and the Contents of Their Blogs.

Motivations	*n*	%	Contents of Blog	*n*	%
Share medical knowledge	116	90.6%	Medical knowledge	115	89.8%
Record my life	88	68.8%	Travel experience	64	50.0%
Provide medical consultations	61	47.7%	Joys and sorrows of job	61	47.7%
Express my feelings	56	43.8%	Literature creations	61	47.7%
Share information non-medical knowledge	51	39.8%	Hobbies or interests	49	38.3%
Marketing myself	38	28.1%	Reading notes	45	35.2%
Communicate with patients	36	29.2%	Delicacies tasting experience	34	26.6%
Communicate with friends	36	29.2%	Family life	29	22.7%
Publish my creation	29	22.7%	Interpersonal relationships	23	18.0%
Contact with relatives/friends	26	20.3%	Finance investment	20	15.6%
Marketing the hospital/clinic where I work	22	17.2%	News critique	18	14.1%
Making friends	13	10.2%	Personal creations	17	13.3%
Others	3	2.3%	Pets	10	7.8%
			Computer knowledge	6	4.7%
			Mood expression	5	3.9%

**Table 3:** The Measurement Properties for Constructs.

Construct	Measurement Item (Indicator)	Composite Reliability (CR)	Loading Value	Other Indicators Loading Value	Mean	S.D.	*t*-Value
Blogging self-efficacy	BSE_1	0.89	0.83	0.13~0.36	3.92	0.82	22.46
	BSE_2		0.92	−0.05~0.43	3.63	0.83	46.16
	BSE_3		0.82	−0.04~0.26	3.34	0.98	18.91
Positive performance outcome expectation	PFOE_1	0.87	0.82	0.09~0.28	3.12	0.84	6.89
	PFOE_2		0.83	−0.07~0.20	3.12	0.98	5.57
	PFOE_3		0.71	−0.02~0.35	2.71	0.99	3.88
	PFOE_4		0.70	−0.20~0.07	3.50	0.93	4.19
	PFOE_5		0.73	0.04~0.30	3.23	0.94	4.65
Positive personal outcome expectation	PSOE_1	0.87	0.74	−0.23~0.11	3.15	1.07	8.23
	PSOE_2		0.75	0.05~0.33	2.85	1.03	7.66
	PSOE_3		0.79	0.01~0.22	2.83	1.05	8.96
	PSOE_4		0.76	0.09~0.41	2.59	0.89	10.34
	PSOE_5		0.74	0.00~0.24	3.23	0.81	8.70
Negative outcome expectation	NOE_1	0.89	0.94	−0.11~0.22	2.38	1.04	5.63
	NOE_2		0.84	−0.03~0.31	2.28	1.03	4.92
	NOE_3		0.79	−0.04~0.46	2.35	0.88	4.69
Satisfaction	SAT_1	0.94	0.88	−0.04~0.55	3.46	0.80	28.80
	SAT_2		0.91	−0.06~0.47	3.54	0.77	45.52
	SAT_3		0.90	−0.01~0.41	3.42	0.77	33.85
	SAT_4		0.87	−0.02~0.42	3.54	0.81	28.54
Continuous intention to blog	CI_1	0.95	0.93	−0.07~0.46	3.92	0.80	45.76
	CI_2		0.95	−0.03~0.50	3.93	0.81	94.33
	CI_3		0.90	0.15 ~0.46	3.77	0.86	26.07

Note: BSE: Blogging self-efficacy, PFOE: Positive performance outcome expectation, PSOE: Positive personal outcome expectation, NOE: Negative outcome expectation, SAT: Satisfaction, CI: Continuous intention to blog.

**Table 4:** Intercorrelations of the latent variables for constructs.

Constructs	AVE	BSE	PFOE	PSOE	NOE	SAT	CI
BSE	0.73	0.85					
PFOE	0.57	0.20	0.76				
PSOE	0.57	0.25	0.03	0.76			
NOE	0.74	0.04	0.15	0.42	0.86		
SAT	0.79	0.40	0.23	0.22	−0.16	0.89	
CI	0.82	0.25	0.23	0.15	−0.18	0.50	0.91

Note: 1. BSE: Blogging self-efficacy, PFOE: Positive performance outcome expectation, PSOE: Positive personal outcome expectation, NOE: Negative outcome expectation, SAT: Satisfaction, CI: Continuous intention to blog. 2. Bolded numbers on the diagonal AVE are values of the square root of the AVE.

## Data Availability

The datasets used and/or analyzed during this study are available from the corresponding author on reasonable request.

## Supplementary Materials

The following are available online at https://www.mdpi.com/article/10.3390/healthcare9080958/s1, Table S1: The measurement properties for constructs.

## Abbreviations

SCT: Social Cognitive Theory; IS: Information Systems; TAM: Technology Acceptance Model; PLS: Partial Least Square; SEM: Structural Equation Modeling; CR: Composite Reliability; SE: Self-Efficacy; BSE: Blogging self-efficacy; PFOE: Positive Performance Outcome Expectation; PSOE: Positive Personal Outcome Expectation; NOE: Negative Outcome Expectation; SAT: Satisfaction; CI: Continuous Intention to Blog; AVE: Average Variance Extracted.
