# Supplementary material for: Factors Influencing Physicians’ Continuous Blogging: A Survey
Source: Healthcare (Basel). 2021 Jul 29;9(8):958. doi: 10.3390/healthcare9080958 (PMC8394209; doi:10.3390/healthcare9080958)
Supplement: Supplementary file 1 [file healthcare-09-00958-s001.zip › healthcare-1291719-supplementary.pdf]

Supplementary Material

Table S1. The measurement properties for constructs

Construct	Measurement item (indicator)	Reference
Blogging self-efficacy	Can present my unique personality<br>Can attracts my audience' interests and attention<br>Can make friends through my blog	[1]
positive performance outcome expectation	Improve my reputation<br>Promote my professional image<br>Increase the number of my patients<br>Provide correct medical knowledge<br>showcase my writings, music, pictures, or other works via my blogs	[2, 3, 4]
Positive personal outcome expectation	Record my life<br>Release my pressure<br>Contact with offline/online friends<br>Expand my social network<br>Feel good	[2, 3, 4]
Negative outcome expectation	Cause negative consequences<br>Cause harmful consequences such as dismissals<br>Feel shame if exposing my weakness	[2, 3, 4]
Satisfaction	Very dissatisfied/Very satisfied<br>Very displeased/Very pleased<br>Very frustrated/Very contented<br>Absolutely terrible/Absolutely delighted	[5]

Continuous intention to blog	continue blogging or updating my blog frequently<br>continue blogging to achieve my expectations<br>continue blogging even though I am busy	[5]

References

[1] Liu X. Online posting anxiety and its influence on blogging: Comparing the US and China. Michigan State University. Department of Telecommunication, Information Studies and Media. 2008.

[2] Quick B. Why doctors blog. 2008. Available from: http://www.healthcentral.com/diabetes/c/110/23419/doctors-blog

[3] Kovic I, Lulic I, Brumini G. Examining the medical blogosphere: an online survey of medical bloggers. Journal of medical internet research. 2018; 10(3): e28.

[4] Liu X. Online posting anxiety: impacts on blogging. Chinese Journal of Communication. 2010; 3(2): 202-222.

[5] Bhattacherjee A, Perols J, Sanford C. Information technology continuance: A theoretic extension and empirical test. Journal of Computer Information Systems. 2008; 49(1): 17-26.
